# New products

**DOI:** 10.1155/S1463924686000317

**Published:** 1986


Journal of Automatic Chemistry/*Journal of Clinical Laboratory Automation*, Vol. 8, No. 3 (July–September 1986), pp. 156–165

### New products

### PITTSBURGH PRODUCT REVIEW

*The 1986 Pittsburgh Conference and Exposition on Analytical Chemistry and Applied Spectroscopy had a record attendance and a well-received technical programme of 1100 presentations. The sponsors are continuing to improve the quality of the programme and are now asking for suggestions for 1987's symposia, short courses and workshops – recommendations should be sent to Joanne Smith, 12 Federal Drive Suite 322, Pittsburgh, Pennsylvania 15235, USA. Joanne Smith is also the person who should receive abstracts for 1987 papers – these are due by August 1986.*

*We are pleased, in the following, to describe some of the new products launched at this year's Pittsburgh Conference.*

### Zymark Corporation

The Zymate II was introduced at the Conference and represents Zymark Corporation's second generation of laboratory robotics.

First introduced at the 1982 Pittsburgh Exposition,the Zymate Laboratory Automation System now offers over 50 different automatically interchangeable robot hands, system modules and interfaces. It is the only laboratory robotics system that can simultaneoulsy operate up to 25 laboratory stations.

The Zymate II features a high-speed robot to improve sample throughput and an updated system controller with enhanced instrument and computer interfacing capability.

To provide higher sample throughput rates, the speed of the Zymate II robot has been doubled and is now variable over a 1 : 5 range. To ensure the reliability of its automated procedures, the fully programmable Zymate II has tactile sensing and collision detection capabilities.

The Zymate II also features applications software called FASTPak (Fast Applications and Start-up Techniques Package). Designed for many common laboratory unit operations, it provides rapid start-up in implementing automated procedures. Using FASTPak's preprogrammed routines, laboratory unit operations such as pippeting, capping, dispensing and weighing can be implemented without having to individually program the detailed steps of these procedures. Programming time is also reduced through FASTPak's

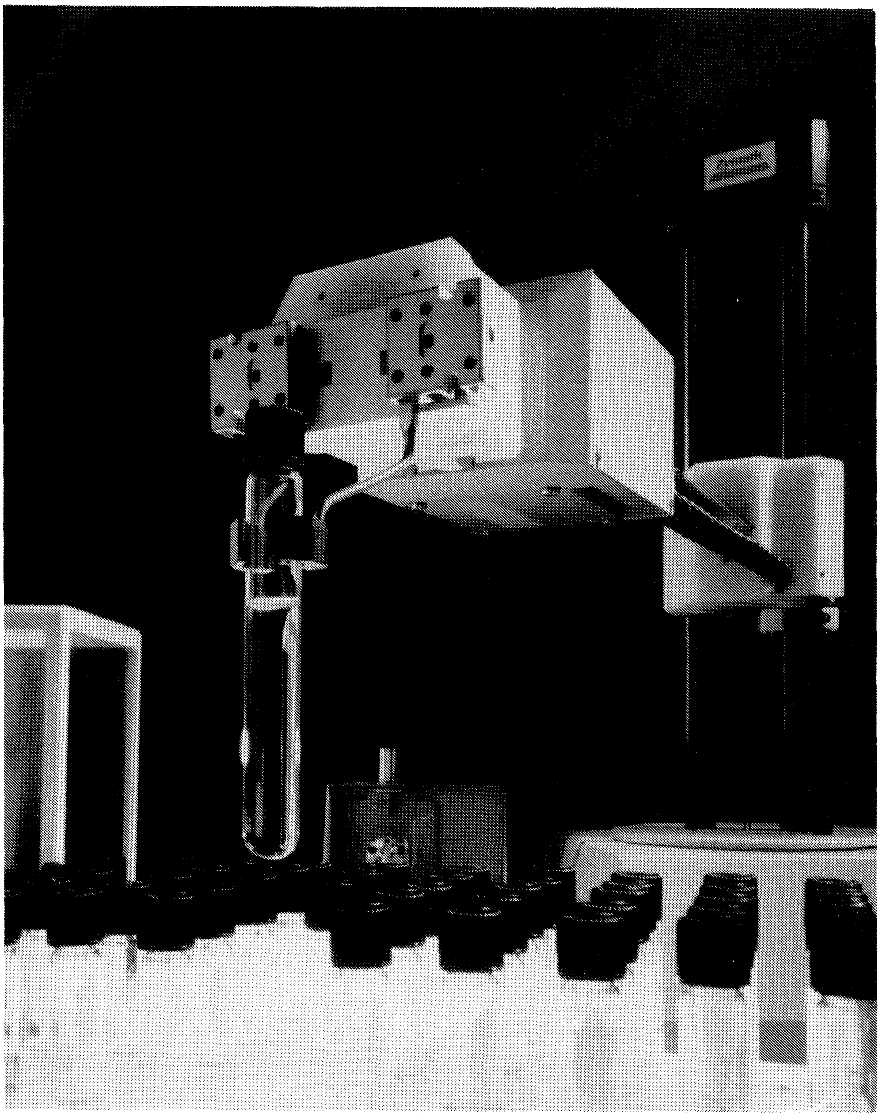

*The new Zymate II(TM) Laboratory Automation System performing an automated analysis.*

*The modular design of the Zymate System guarantees its future expandability. Retrofit packages are available which allow all existing Zymate robots to be updated to the Zymate II configuration. Laboratory automation systems incorporating the Zymate II are priced between $35 000 and $40 000. Optional features such as instrument interfaces and the bar code reader are available at additional cost.*

automatic serialization and sample scheduling capability.

Custom and standard software packages are also available with the new system for laboratory integration of robotics, analytical instruments and computers. In addition, the Zymate II Controller provides a wider range of computer and instrument interfacing capabilities. An improved Remote Control/Computer Interface is available with the system, permitting the Zymate II to be operated either by a host computer or from the Zymate Controller. This frees any auxiliary system connected to the computer for simultaneous data processing or report generation.

'Plug-in' interfaces for analytical instruments have been developed for the Zymate II, allowing instruments to be operated directly from the Zymate Controller. Instruments for which dedicated, preprogrammed interfaces have been developed include: Hewlett-Packard gas chromatographs, liquid chromatographs and UV/Vis spectrophotometers; Mettler balances and titrators; and Beckman spectrophotometers. Custom interfaces allow a wide range of other analytical instrumentation to be integrated into the Zymate II. A optional bar code reader is now available as a standard Zymate module, providing positive sample identification during unattended operation. Applications reliability is enhanced as bar coded sample labels can be read and decisions made as to which analytical procedure is to be performed for each sample.

*The Zymark Corporation can be contacted at Zymark Center, Hopkinton, Massachusetts 01748, USA. Tel.: 617 435 9501.*

### Becker

E. M. Becker & Co. demonstrated a CHN analyser which is about half the price of competing equipment—the analyser employs a new PTV concept. Sample weights from 1 to 200 mg can be used and positive 100% instantaneous combustion produces results that minimize nonhomogeneous sample effects.

*Details from EMBCO, PO Box 119, Bala Cynwyd, Pennsylvania 19004, USA. Tel.: 215 483 4225.*

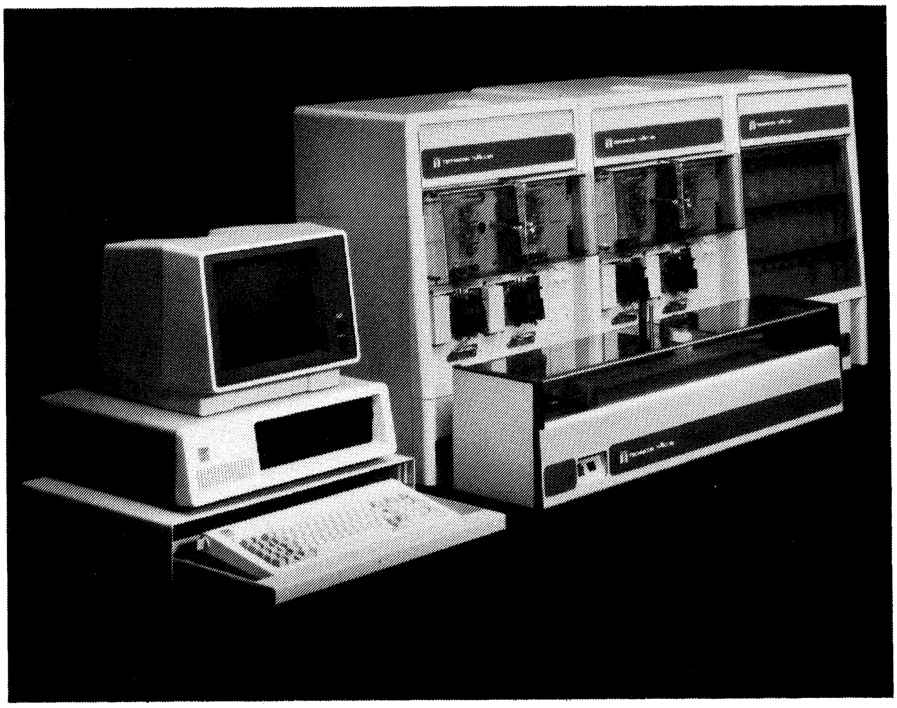

*TRAACS 8000 introduced at Pittsburgh in March 1986.*

### Technicon

Technicon Instruments Corporation's Industrial Systems Division launched the TRAACS 800 (Technicon Random Access Automated Chemistry System) at the '86 Pittsburgh meeting. According to a Technicon spokesperson: 'the availability of this totally computer-controlled system advances continuous-flow analytical technology to a new high level of automation . . . with equivalent advances in precision and accuracy. Its compact design and performance criteria were specifically established with today's laboratory in mind'.

Under computer control, the Technicon TRAACS 800 system can automatically initiate operation, sequence reagents, set base-line and gain automatically, and generate real-time as well as corrected results. The random access sampling system accommodates up to 120 sample cups and utilizes a patented 'pecking-at-the-source' feature to improve sample throughput and wash characteristics. The four-channel version is capable of testing four chemistries simultaneously; the dual-channel handles two chemistries at the same time.

Samples which exceed the range of analysis are automatically diluted and reanalysed without operator intervention. By utilizing the automatic reagent sequencing feature, and the multi-test chemical manifold, change-over from one chemistry method to another is automatic. High sample analysis rates, with improved accuracy and precision, are achieved through combination of electronically controlled bubble injection, bubble-through-the-flow-cell, and a new sampling technology.

*For additional information on the Technicon TRAACS 800 system, write to The Manager, Wet Chemistry Systems, Technicon Instruments Corporation, Industrial Systems Division, 511 Benedict Avenue, Tarrytown, New York 10591, USA, or 'phone 914 333 6142.*

### Isco

Two gradient chromatographs were announced by Isco: economically priced, the systems feature a programmable pump that changes flow rate based on either time or volume delivered; and a choice of either a single-pump binary/ternary gradient programmer or a computer-controlled, two-pump gradient system with data management. A detector with a noise level of only $2 \times 10^{-5}$ A, 8,000 h lamp life, and flow cells for any application from microbore to industrial prep LC are also included.

Both chromatographs are complete with all extras including column valve, fittings, and recorder or printer.

*More information from Isco, Inc., PO Box 5347, Lincoln, Nebraska 68505, USA.*

### Dionex Corporation

The Series 4000i makes it possible to do continuous gradients. The power of HPLC is also expanded by problems that chromatographers have struggled with for years – the analysis of polar and ionic organic compounds.

The Series 4000i delivers this capability with a new high-pressure gradient pump, patented IonPac polymer and silica columns, and Dionex's proprietary MicroMembrane Suppressors – these are non-metallic to eliminate corrosion and metal contamination.

*Details from Dionex Corporation, PO Box 3603, Sunnyvale, California 94088–3603, USA. Tel.: 408 737 0700.*

### COSA

The Mitsubishi GT-05 is able to set its own titration parameters. The program does method development – it is only necessary to determine the electrode pair and add the sample.

The GT-05 can be pre-programmed for routine titrations. A dot matrix printer gives a complete permanent record of each analysis. The classical titration curve and the first derivative including marked inflection points, end point volumes, calculated results and titration parameters are clearly indicated. The titration data remains stored in the memory after the titration is completed and can be recalculated and displayed with modified parameters.

*For more information contact COSA Instrument Corporation, 70 Oak Street, Norwood, New Jersey 07648, USA. Tel.: 201 767 6600.*

### Tracor Instruments

A new system for determining sulphur content and a range of high-performance image analysers were launched by two of Tracor's companies at Pittsburgh.

*Sulphur*

The Tracor Atlas Model 825R-D/856/1003 Total Sulfur Analyzer provides fast, accurate and economical measurements of sulphur content from low p.p.b. to high percentages. The system improves laboratory versatility by detecting and analysing $H_2S$ and Total Sulfur for nearly any gas or liquid sample and for most solids.

*Details from Tracor Atlas at 9441 Baythorne Drive, Houston, Texas 77041-7709, USA. Tel.: 713 462 6116.*

*Image analysis*

Tracor Northern offers two distinct systems for high-performance image processing and analysis. The TN-5700 System (including the TN-5500 X-ray Analyzer) provides the only image processing system which totally integrates X-ray microanalysis. When X-ray analysis is not required (such as with optical or transmission electron microscopes), the TN-8500 offers image processing at the same high level of performance.

Both systems feature image acquisition using the Kalman technique which has been proven superior to other frame averaging methods. Both systems possess a complete selection of grey level filters, binary filters, grey level transformations, colour scales, image mathematics, Boolean logic and image editing. Feature analysis provides geometric information including area, perimeter, average diameter, length, width, feret diameters and centre, and orientation. Innovative use of a pipeline image procesor (PIP) allows grey level filtering and image mathematics to be performed on a 1024 × 1024 image in a second or less.

*Tracor Northern can be reached at 2551 W. Beltline, Highway, Middleton, Wisconsin 53562, USA. Tel.: 608 831 6511.*

### Link Systems

The XR200 Series, new from Link Systems, was promoted as handing solids, liquids and powders directly performing simultaneous multielement analysis; analysing from PPM to 100% without dilution; performing unequalled rapid qualitative analysis on 80+ elements; having flexible software for ease of use; being a modular system which can be tailored to users' needs. The XR200 Series is in the same price range as AA.

*More information from Link Analytical, 240 Twin Dolphin Drive, Suite B, Redwood City, California 94065, USA. Tel.: 415 595 5465.*

### Pierce

The new Reacti-Therm III Heating and Heating/Stirring Modules offer increased sample capacity and versatility in dry block heaters. The new design provides for the use of three Reacti-Block Aluminium Blocks to triple sample preparation capacity. The units are ideal for high temperature derivatizations, digestions and protein hydrolyses due to their expanded temperature range of ambient to 200 °C. The Reacti-Therm III Heating/Stirring Module has a built-in stirrer and provides smooth, uniform reactions and dissolves sticky residues. A versatile 27-port evaporator, the Reacti-Vap III, facilitates sample isolation and concentration.

The original Reacti-Therm Heating and Heating/Stirring Modules are available for single block requirements. Stirring and evaporating capabilities are available with these units. Their temperature range is ambient to 150 °C.

*Details from Pierce Chemical Company, PO Box 117, Rockford, Illinois 61105, USA.*

### Fisher

The Maxx-5, a robot, was launched with other products at Pittcon. Maxx-5 works well suspended upside down from a track. Hence, where his conventional brothers are limited to a circular work area within arm's reach, the Maxx-5 robot serves in a number of work areas as he moves along his track.

Also new at Pittsburgh were:

*PrepSep-R*
The first solid-phase extraction columns designed specifically for robotics.

*Model 400*
A new approach to digital burettes, a convenient compact, for use with the Computer-Aided Titrimeter or CAT, that extends resolution 0·001 mL, *independent of titration mode.*

*Blue Chips*
Custom chips for CAT. They automate 25 crucial analytical and QC tests in chemical, pulp and paper, pharmaceutical oil, petrochemical, textile fibre, and food technology laboratories.

*VersaBath-S*
The first shaking-waterbaths with microprocessor control. All parameters are set buy keyboard.

### LC/EC wall-jet cells

A range of wall-jet cells for HPLC electrochemical detection is now available from Severn Analytical. The cells form part of the Coulochem system and combine a coulometrically efficient porous graphite screen electrode with an interchangeable wall-jet electrode. This enables reactions to be accomplished on surfaces such as nickel, gold, platinum, glassy carbon and gold/mercury amalgam, thereby widening the range which can now be analysed to include compounds such as benzodiazepines, nitrosamines, organic explosives, aromatic nitro compounds, nitramines and nitrate esters. The cells have a solid state reference/counter electrode which provides rapid stabilization times, and all electrodes are repolished in one simple operation.

*Severn Analytical is based at 36 Brunswick Road, Gloucester G11 1JJ, UK. Tel.: 0452 20306.*

### RTQC and blood gas analysis

Beckman offer a 'Real Time Quality Control System' to monitor blood gas analyses with Pathway pH/blood gas controls. Pathway controls are stored at room temperature; therefore no temperature equilibration is required prior to use. There are four clinically essential levels: Acidosis, Normal, Alkalosis and Elevated Oxygen which are available in colour-coded vials. Values are provided for most blood gas analyses and the aqueous base of Pathway controls deters protein build-up. They maintain stability for 18 months and once the vial is opened stability is assured for 60 s.

The system carries out all quality control calculations – with instant reporting on acceptability of results. Users are alerted to bias, trends, rotation of curvature or non-linearity, and potential problems can be detected even before a control is beyond acceptable limits. Monthly data can be summarized at the touch of a button and quality-control charts printed as required.

*For further information contact Beckman Ltd, Progress Road, Sands Industrial Estate, High Wycombe, Buckinghamshire, UK. Tel.: 0494 41181.*

### Applications bulletin

An applications bulletin from Nermag demonstrates the use of their low-cost R10-10T mass spectrometer combined with the SIDAR data system to the quantitative determination of a drug in human plasma.

*Copies are available from Dorand Electronics Ltd, Allens Lane, Hamworthy, Poole, Dorset BH16 5DA, UK. Tel: 0202 622006.*

### Touch screens

A new hard surface coating provides additional protection against scatching for Elographics' E270 and E274 Touch Screens when used in hostile environments, such as for process control terminals on the factory floor. This transparent hard surface coating is applied to the front surface of the touch screen's contact sheet, and is available with an anti-glare effect.

The membrane cover sheets, themselves, provide important protective features. The plastic cover sheet on the resistive membrane touch screen protects the thin-film coating on the glass from wear or abuse during customer use. Unprotected, thin-film coatings, as with capacitive touch screens, are susceptible to wear and accidental or vandalizing scratches which destroy the touch screens' linearity.

*Details from Dicoll DataSystems Ltd, Bond Close, Kingsland Estate, Basingstoke, Hampshire RG24 0QB, UK. Tel.: 0256 461551.*

### Single/dual channel computing integrator

Trivector Systems International have complemented their range of integrators with a low-cost product called TRIO. Both hardware and software are designed with the chromatographer in mind. The software is driven through user-friendly menus. TRIO offers one or two channel, synchronous or asynchronous data collection from any chromatography through a high accuracy V/F convertor.

The incoming chromatograms can be shown in real time on the graphics display offering exceedingly high resolution of 720 × 480 pixels. The TRIO is highly flexible in scrolling and magnifying data in real time or post run mode. The post run display also incorporates base-line and peak identification including graphical comparisons. TRIO uses a digitization technique which integrates incoming signals continuously using a blend of hardware and software. The data is stored as a series of integrals, the time period of each being dependent on the width of the peaks in the chromatogram.

Each integral is free from mains noise and has a resolution of $1:10^6$ for each second of integration. This technique has been well proven throughout the lifetime of the existing TRILAB range. An additional feature is that all data points are stored as differences, each data point occupying only the minimum required for its magnitude. This reduces the storage required for each chromatogram to about one half of that otherwise necessary, without affecting the resolution of stored data. TRIO contains three discrete microprocessor systems, with the analysis processor running at 6 MHz.

The raw data is stored within the 200K of internal RAM memory and then subsequently analysed using one of 10 method files that are stored within the 8K of battery backed-up RAM. Many of the advanced features of the TRILAB 2000 and 3000 are available, including the peak-detection algorithm together with the unique graphical base-line definition routine. All standard modes of analysis are available from normalization to internal standard calibration with varying response factors. Results and chromatograms (including base-line) can be printed to either plain paper graphics printer type 2014, inkjet printer type 2023 or a thermal printer/plotter type 2012. For archiving, the raw data and results can be stored on the dual 3·5 in floppy disk option. The data can also be passed to the TRINET Laboratory Information Management System by a state-of-the-art, fibre-optic coupler, capable of transmitting data at 768 K baud.

There is also the novel possibility to plug-in optional library ROMS. Each ROM contains 128 K of useable program storage. A number of programs may be supplied in each ROM and an internal index within the ROM enables TRIO to access each program as required. BASIC is available and GPC, Amino Acid and special calibration packages are being produced.

*Trivector Systems International can be reached at Sunderland Road, Sandy, Bedfordshire SG19 1RB, UK. Tel.: 0767 82222.*

### New automated coagulation laboratory

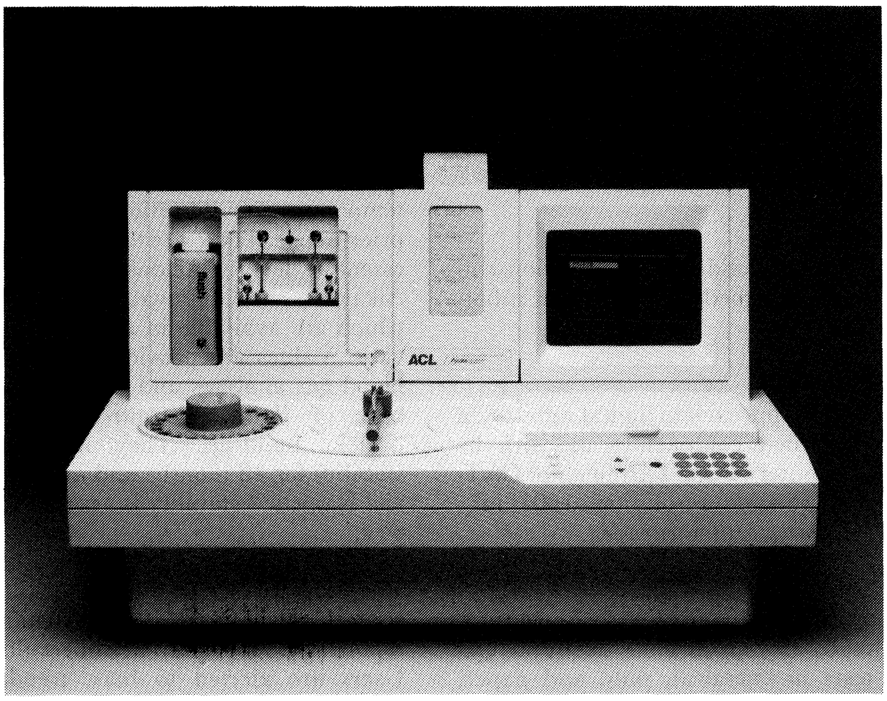

*The ACL system from Instrumentation Laboratory. The system's multilingual software is available in English, French, German, Italian or Spanish. An on-board, dedicated QC program helps assure consistent accuracy and precision. This includes daily data storage for up to one month, plus the display and plotting of QC data and the calculation of relevant statistics.*

The ACL Automated Coagulation Laboratory from Instrumentation Laboratory is essentially two systems in one, performing both clotting and chromogenic assays. It performs all traditional clot-timing analyses, plus colorometric assays using enzymatic substrates.

Its multichannel capabilities allow not only routine screening, but also the special assays necessary to complete a diagnosis.

Because of its exceptional analytical precision and microcentrifugation technology, the system uses one-half to one-fourth the reagent volumes required in other systems. Only 50 to 100 microliters of reagent are needed for any of the tests, about half that required by other systems; precision is high enough to avoid testings. These two improvements significantly lower the cost per test.

The ACL provides quicker coagulation results than other systems. It offers fully automated calibration, reagent and sample loading, analysis, printing and quality control, eliminating such tedious steps as performing manual dilutions.

Up to 18 samples can be analysed simultaneously – in 5 min for PT (maximum throughout, 216 samples/h simultaneously – in 5 min for PT (maximum throughput, 216 samples/h), or a maximum of 9 min for APTT (120 samples/h). As many as 60 samples may be analysed per hour for PT + FIB/APTT. This amounts to stat speed on batch runs.

Fibrinogen can be measured routinely with each PT determination, with fewer manual steps and less reagent.

Concentrations of plasminogen and alpha-2 antiplasmin are also determined to simplify the investigation of fibrinolysis.

A non-volatile memory protects calibration parameters.

Extensive instrument self-diagnosis programs help to assure the correct performance of all automated functions.

*For more information 'phone 617 861 0710. Or write to Instrumentation Laboratory, International Division, 113 Hartwell Avenue, Lexington, Massachusetts 02173, USA.*

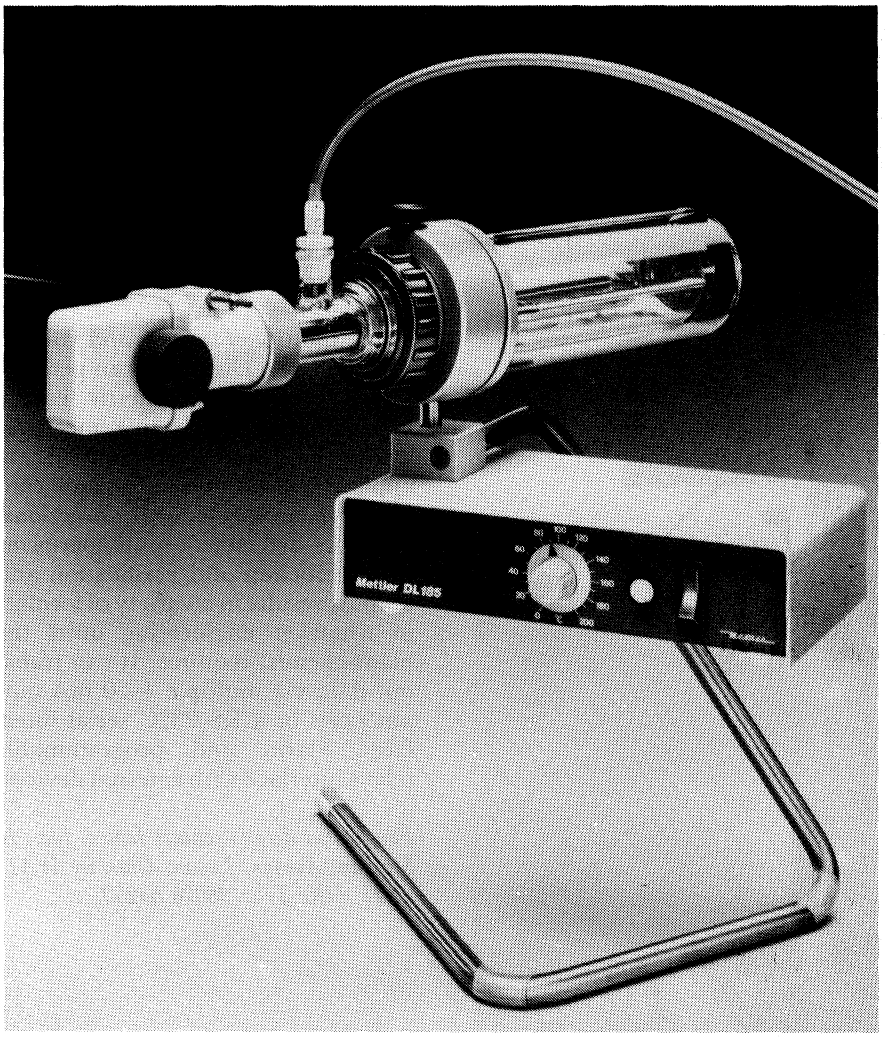

*With the Mettler DL185 drying oven, even strongly bound water, as well as traces from stubborn samples such as plastic granules, can be extracted by heating. The thermally extracted moisture is led to the titrator with dry purge gas. The DL185 is also well suited for samples which react with the titrating solution – vitamin C for example.*

*Loading is easy: samples are inserted into the large metal crucible through the standard ground connecting piece, or after the locking device has been opened. The metal crucible is then inserted in the heated section of the glass tube by means of the control handle. The preselected temperature can be continuously adjusted up to 200 °C. This corresponds approximately to the actually attainable sample temperature and is one of the reasons why the results are so reproducible. Another reason is the stream of dry purge gas which prevents moisture from penetrating the oven, even when the usually closed system is opened. The oven and the titrator can be standardized with plain water.*

*Mettler can be contracted at CH 8606 Griefensee, Switzerland*

### Water box

During the last few years water and environmental laboratories have both shown a need for the automation of pH and conductivity measurements. Radiometer having 50 years' experience in both measurements have combined those of their latest microprocessor-controlled instruments the PHM83/85, CDM83 and the SAC80 through an interface known as the 'water box'. This allows the user to automate up to 20 samples at a time for pH, conductivity and temperature and output the results onto the Radiometer PRS12 Alpha Numeric Printer, or onto a computer.

The modularity of Radiometer instruments also allows for the system to work with ISEs using the ION83 or 85 in place of the pH meter. The ultimate combination will allow the user to measure pH, conductivity and then titrate the sample. This uses the MTS titration system in place of the pH meter or the ION meter.

*Details from V. A. Howe & Co. Ltd, 12–14 St. Ann's Crescent, London SW18 2LS.*

### Pipetting

The Accuflex-TP pipetting station can be operated by either the IBM personal computer or the BBC microcomputer and is supplied complete with software disk. The program is written in BASIC and can be easily modified to suit the user's requirements. The computer can store in memory the protocols for different immunoassays so that the time for setting up complex assays is dramatically reduced. The Accuflex-TP can accept a wide variety of sample and receiver vessels from micro-titre plates through to standard RIA tubes and larger bottles.

The station can be easily programmed to accept commercial RIA kits, such as the Corning Magic range, without any need to transfer reagents from their original containers.

The sample volume range is from 2 µl to 2 ml, the dispensing volume from 2 µl to 20 ml and the diluent volume from 2 µl to 10 ml with an accuracy of ±1% or 0.1 µl. The Accuflex can dispense at a rate of 1400 aliquots/h.

Accuflex is designed so that the delivery tip remains stationary while the carrier holding the sample and receiver vessels is moved. This avoids inaccuracies caused by moving liquid lines.

The Accuflex-TP, which is competitively priced, should be useful in any situation where there is repetitive transfer and/or dilution of small volumes of liquid.

*Further information from ChemLab Instruments Ltd, Hornminster House, 129 Upminster Road, Hornchurch, Essex RM11 3XJ, UK. Tel.: 04024 57011.*

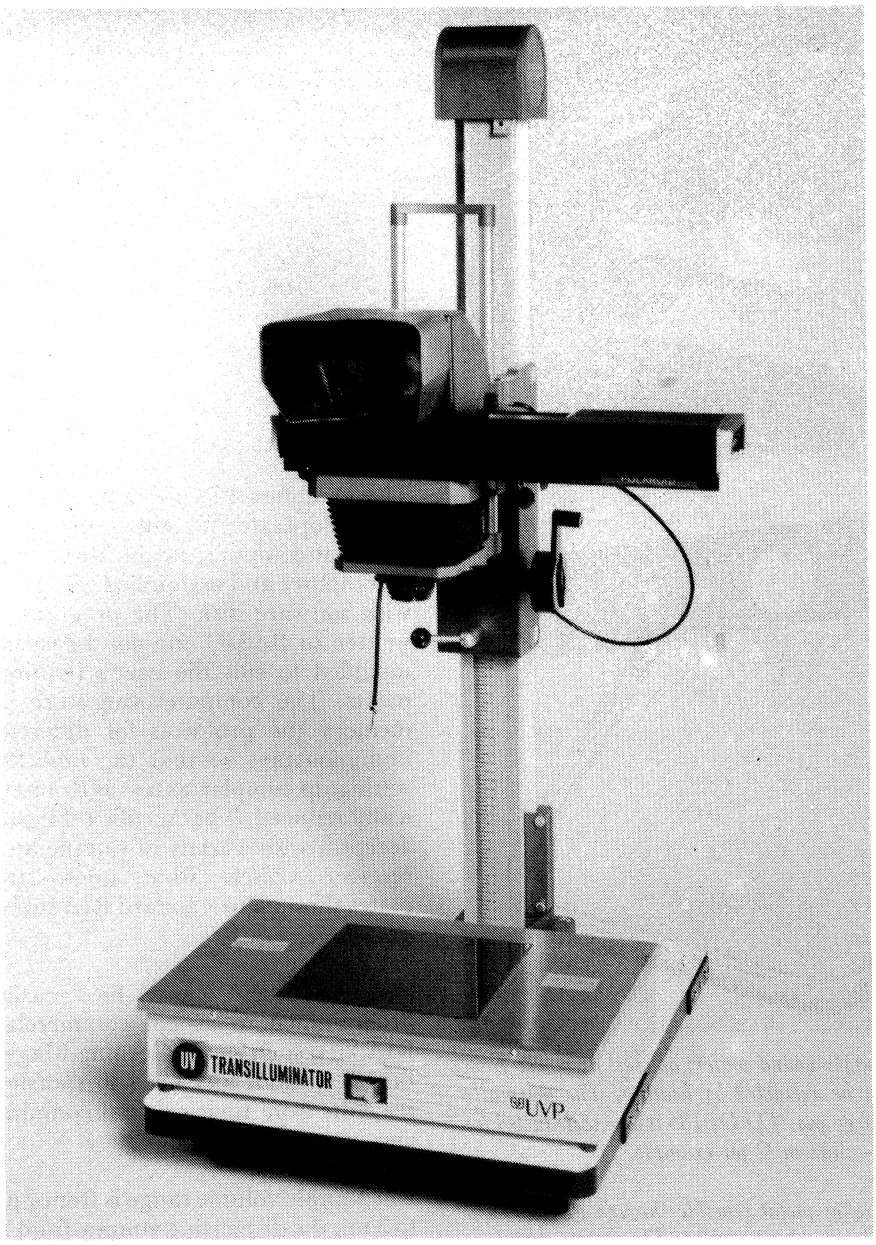

*Ultra-Violent Products is now offering a comprehensive supporting range of gel preparation and viewing equipment. Options are sufficiently wide to enable customers to build electrophoresis preparation kits to meet their own specific needs. A choice of alternative Polaroid camera equipment – sheet, film or new 35 mm slide – includes a wide selection of stands, hoods, films, film holders and other necessary items of photographic material. Details from Ultra-Violet Products, Science Park, Milton Road, Cambridge CB4 4BN, UK. Tel.: 0223 355722.*

### Sampling in on-line process analysis

In on-line analysis difficulties are often experienced in introducing a representative sample to the analyser. Ionics have developed a new sample addition valve for their Model 3000 Digichem process chemical analyser which is claimed by the company to overcome such difficulties. The Digichem is used in a variety of industries including petrochemicals, electro-plating, pulp and paper, chloro-alkali, waste water and air pollution control. Built for 24 h continuous operation it not only reduces the operator's exposure to hazardous materials but also increases the repeatability of the analysis.

The new valve allows the process sample to be continuously recycled. Designed for reliability and easy maintenance, it consists of only one moving part, including the actuator. This is a shuttle, which, as it changes position, captures a precise sample volume. The sample is then washed by an appropriate diluent into the Digichem's reaction cell. The system contains no peristaltic pumps nor tubing prone to plugging or failure.

Using multiple reagents and various detectors, the Digichem can perform a wide variety of titrimetric, colorimetric and ion selective measurements, on single or multistream applications. A microprocessor controls electromechanical functions, monitors detector outputs, performs data reduction and verification and outputs results in a variety of formats in whatever engineering units the plant chemist requires. It can transmit data via multiple 4–20 mA output ports or a RS 232C serial interface. Alarm and programmable relays interface with external devices.

*For further details contact Ionics, Inc., 10 Statham Avenue, Lymm, Cheshire WA13 9NH, UK. Tel.: 08926 63259.*

### Cardiac drug research

Various aspects of cardiac research projects benefit from a technique used by pharmaceutical company SmithKline & French of Welwyn. Using Beckman's Paragon agarose gel electrophoresis system, the team at SK&F require a high degree of sensitivity in a method for detecting early cardiac damage involving isoenzyme studies.

The Paragon system offers a broad linearity range for isoenzyme detection and the new template applicator, which is a special feature of the Paragon package, allows specimens to be screened with minimum concentration, providing unparalleled sensitivity. These advantages are of particular value to the SK & F team which is led by Head of Clinical Pathology, Richard Barrett. Mr Barrett points out that the agarose gel medium is itself very suitable for their purposes and that, with the Paragon system, a permanent record of selected LDH results can be made and easily filed for future records and reference, as well as comparisons.

A poster on the studies was presented by Richard Barrett at the most recent meeting of the Animal Clinical Pathologists at Warwick, UK.

*Beckman can be contacted at Progress Road, Sands Industrial Estate, High Wycombe, Buckinghamshire, UK.*

### Interactive gas chromatography system

A new gas chromatography system from Hewlett-Packard combines the power of the dedicated HP 5895A GC workstation with the proven chromatographic precision of the HP 5890 GC instrument. The system has been designed to meet the complex needs of laboratories involved in GC methods development and modification. The instrument's set-up parameters and its operation are controlled from the workstation, which also provides dual-channel data acquisition, disk storage, a full range of data reduction manipulations and report generation.

The system can be used to calculate retention indices and to search an online library data-base for potential matches. This capability, combined with recent HP advances in column technology and with the thermal stability of HP's column ovens, delivers a new level of qualitative information to the gas chromatographer. Commerically available data-bases may be used, or users can develop their own libraries of retention indices.

The workstation has a multiple windowing facility which allows chromatograms to be displayed simultaneously on the screen and directly compared. Addition and subtraction routines may be performed and then displayed graphically; and chromatograms may be overlayed in different colours.

A zoom feature allows small segments of the chromatogram to be expanded to fill the entire screen, thus permitting more detailed examination of specific peaks. During a run, earlier portions of the chromatogram may be recalled and reviewed on screen, with a single soft key used to return to the real-time plot.

The advanced integration algorithm of the system is optimized for most situations. Interactive software makes it a simple matter to alter the integration parameters as required. Mass storage of data (up to 55 Mbytes) allows reintegration and replotting to be carried out without having to reinject the sample. Methods and results may, of course, also be stored.

The system's workstation is one of a series newly introduced by Hewlett-Packard and is based on the HP 9000 Series 300 technical computer and a core set of chromatographic software tools. These workstations are currently being made available with HP's range of gas and liquid chromatographs and chromatography/mass spectrometry systems. All share a common approach to method definition, data acquisition, post-run data processing and sequencing for automation. The workstation is supplied with a monochrome or a colour monitor, Winchester disk drive and HP ThinkJet printer.

*Enquiries to Tina Mears, Analytical Instrumentation, Hewlett-Packard Ltd, Miller House, The Ring, Bracknell, Berkshire RG12 1XN, UK. Tel.: 0344 424898.*

### OSCA chemical sensing group

The Optical Sensors Collaborative Association (OSCA) has now formed a Chemical Sensing Group to coordinate OSCA's activities in this field. The objectives of the Group are to assist the development of chemical sensing technology through the application of electro-optic and fibre-optic techniques, to support research in chemical sensing complementary to work currently in progress elsewhere, and to provide the OSCA membership with information on chemical sensing generally.

The main field of the Group's interest is in gas sensing both at point measuring locations and also over distributed areas, particularly for concentrations of methane or other flammable gases. Carbon monoxide in air and in flue gases and, generally, toxic gases, industrial gases and pollutants are also of interest, together with measurement of dissolved species in liquids and also bio-sensing.

OSCA welcomes enquiries from organizations which might like to share in the overall R & D programme, receive copies of reports from previous projects covering surveys, laboratory and demonstration projects, multiplexing and system studies, and participate in the patent and current awareness information services.

*For further information please contact Mrs P. West, OSCA Manager, Sira Ltd, South Hill, Chislehurst, Kent BR7 5EH, UK. Tel.: 01 467 2636.*

### New thermoplastic tubing for peristaltic pumps

A range of Esco thermoplastic rubber tubing is now available from Sterilin. Called Plescoprene, the new tubing has exceptional resistance to mechanical action and is ideal for all peristaltic pump applications. Manufactured from food grade material, Plescoprene offers good environmental resistant to heat, cold, ozone and weathering, and has chemical resistance properties similar to neoprene grades of synthetic rubber. It is suitable for continuous running at temperatures of up to 125 °C, and for short periods (one week) at 150 °C. Repeated sterilization with steam or ethylene oxide poses no problems.

*More information from Sterilin at Sterilin House, Clockhouse Lane, Feltham, Middlesex TW14 8QS, UK.*

### Analysis and quality control literature

An *Application Study* has recently been completed by Paar Scientific and is available free of charge to interested readers in industrial analysis and quality control operations. This latest in the Paar series of study sheets illustrates the application of several Paar density meters to analytical and quality control techniques in the laboratories and production line of a leading toiletries manufacturer – Elida Gibbs Ltd.

There are three main areas of work where the Paar DMA 46 digital density meters in particular are found of special benefit to Elida Gibbs. In the company's Product Control Laboratories for example, they are used to test samples of shampoo and aerosols at raw material and final product stages. In the Special Analytical Laboratory they are used as a support measure for various stages of the stringent quality assurance operation.

The portable version DMA35 is also regularly in use for lineside checking, for raw material tests, including perfumes, and for inspection personnel to carry around for sample testing during production.

*Copies from Paar Scientific Ltd, 594 Kingston Road, Raynes Park, London SW20 8DN. Tel.: 01 542 9474.*

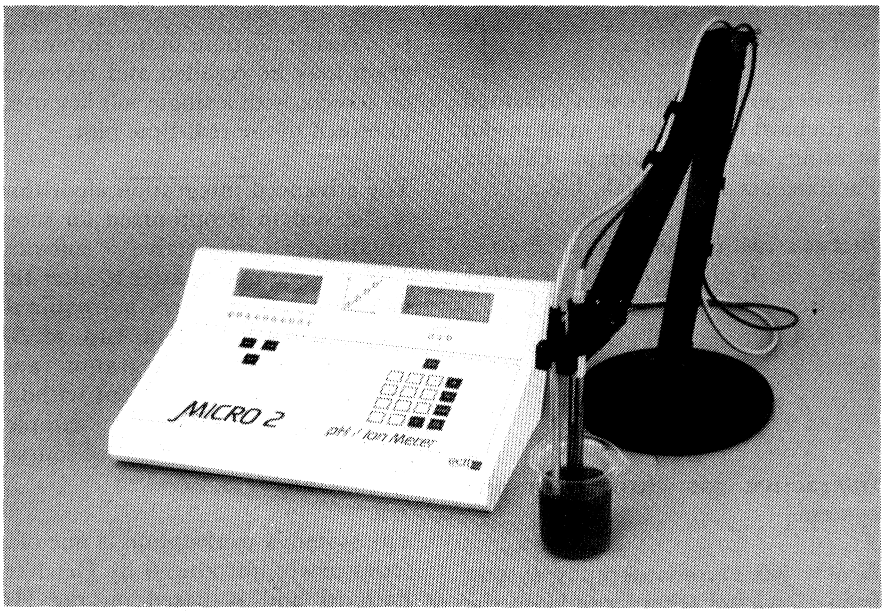

*Micro-2 – Courtcloud's pH/ion meter. Priced at around £850, the instrument has three input sockets for electrodes which can be calibrated independently and addressed from the front panel. There are two LED displays, one of which shows continuously the temperature measured by the standard temperature probe. This is used to make automatic temperature compensated measurements. The other display, accurate to 0.1 mV, shows the measured value which may be pH manual mode, pH ATC mode, mV relative, ion concentrations in ppm, Molar or px units. In pH and Ion modes, the electrode slope is calculated and can be displayed to monitor the performance of electrodes.*

*The sophisticated software has many 'input-check' features which prevents incorrect or inappropriate values being entered. An audio feedback informs the user that an input or command has been rejected. A 'check' mode is available to recap any calibration data in the memories while a unique 'key-board lock' feature allows all non-essential keys to be disabled after calibration is complete. This prevents any inadvertent data entries being made. Battery back-up retains calibration data on 'power off' for up to seven days.*

*Details from Courtcloud Ltd, Lorne Road, Dover, Kent CT16 2DR, UK. Tel.: 0304 213555.*

### Enhanced graphics capability on IBM PC infra-red data-station

Spectrafile IR, software that turns the IBM PC or approved compatible into an infra-red data-station, now supports high-resolution colour graphics. This will be of particular interest to spectroscopists in quality control who wish to match samples versus standards quickly and unequivocally. The new colour option doubles the normal IBM colour resolution to 640 × 400 pixels.

Another recently introduced graphics feature is the ability to define and magnify up to nine discrete wavenumber regions or 'windows' within a spectrum. The user may save sets of windows to disk, reloading them subsequently whenever required.

Again with QC spectroscopists in mind, Spectrafile has been equipped with routines for creating and searching libraries of full deresolved spectra. This system has the advantage that library spectra may be recalled immediately after a search for a visual check. Search 'hit quality' values serve to quantify the absolute difference between the unknown spectrum and each 'hit' from the library. This could easily form the basis of a preliminary pass/fail test for incoming samples.

*Details from Heyden & Son Ltd, Spectrum House, Hillview Gardens, London NW4 2JQ. Tel.: 01 203 5171.*

### X-ray microanalysis system

A low cost X-ray microanalysis system has been developed by Link Systems Ltd. The company is promoting the PXA/1 system to cost-conscious industrial and educational users, taking advantage of the proven 16 bit system architecture and attractive ergonomics of the Apricot PC.

PXA/1 is a complete system, including Si(Li) detector and all operating software. It utilizes pulse processor technology and operating routines to those in more sophisticated systems. However, a major advantage is that the system uses the industry standard MS-DOS operating system – with 3·5 in microfloppy disks – and can be used for other purposes – word processing, spreadsheet analysis of results, or running other specific educational software packages.

The pulse processor and its interface to the microcomputer are housed in a single unit; the interface is designed in such a way to preserve data integrity with minimum system dead time. The analyser is menu driven utilizing a high resolution colour display and remote keyboard linked to the computer by an infra-red beam. Major features include a 2 ×

2 K data memory with 32 bit capacity, ability to store data on disk, bar and dot display for spectrum comparison and element identification by KLM markers. Multiple ROI windows may be viewed, calibration and acquisition parameters displayed and the display may be operated in a split-screen mode.

*More information from Eric Samuel (Link Systems Ltd) on 0494 442255.*

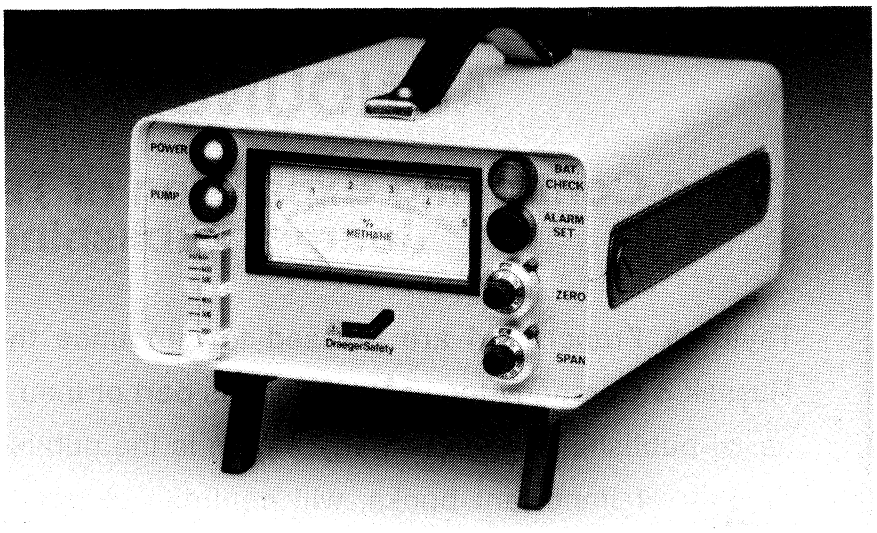

*A range of infra-red gas analysers has been introduced by Draeger Safety, Blyth, Northumberland, for applications requiring a tough, portable, battery-powered gas analyser for the measurement of carbon monoxide, carbon dioxide, methane and other hydrocarbons. Called the Infralarm, the system is fitted withan audible alarm that is internally adjustable across the range of the instrument, and has an internal mute switch. With optional probes and filters the Infralarm provides a low-cost analyser for use in furnace atmospheres, stacks and vehicle engine exhaust measurements. Without the probes, applications exist in agriculture, controlled atmosphere chambers, power generation and the nuclear industry as well as in other areas. Details from Draeger Safety, PO Box 4, Blyth, Northumberland NE24 1HA, UK. Tel.: 0670 352602.*

### 'Techniques in Graphite Furnace Atomic Absorption Spectrophotometry'

This new softback from Perkin-Elmer contains 224 pages of valuable information specific to Stabilized Temperature Platform Furnace (STPF) technology. STPF is a concept that combines hardware and parameter selections to provide more accurate and reliable data with reduced method development time. The STPF concept includes use of the L'vov Platform in a pyrolytically coated tube, peak integration with base-line offset correction, maximum power heating, stopped internal gas flow during the atomization step, matrix modification and efficient background correction.

The manual is organized into two sections. The first part contains general information about the Stabilized Temperature Platform Furnace concept and graphite furnace method development.

The second section contains specific instrumental recommendations for each element for either Zeeman or continuum source background correction. Information on wavelength, slit, pretreatment and atomization temperatures, injection location, matrix modifier, and characteristic mass, are supplied to aid in graphite furnace operation.

The book costs £27.00 (Part Number 0993–8150).

*Orders to Perkin-Elmer Ltd, Post Office Lane, Beaconsfield, Buckinghamshire HP9 1QA, UK. Tel.: 04946 6161.*

### Intelligence for measurement control

Drew Scientific has developed an intelligent control and data logging system for such industrial applications as brewing, food, chemicals, water and pilot plant laboratories. The system provides a comprehensive software suite, low-cost entry, 'building block' method with board level growth principles.

The building block approach makes use of distributed plant interface computers linked to a master computer which provide the overall intelligence to the operations. Each distributed interface computer offers up to 360 input or output channels with control signals or measurements from temperature, pressure, position and speed sensors, load cells or strain gauges. Additionally, the system incorporates SPL—the Sequence Programming Language—which removes the need for operator training and gives facilities for simple alteration of process commands. SPL can support and control up to five distributed interface computers and allows flexible control of user definable multiple sequence operations and profiled set-point control.

A comprehensive alarm system is available for continual checking of plant activities and can be made dependent on measurement levels or digital signal inputs.

Collecting data is simple, logging digital or analogue data on a time and rate of change basis. Once collected onto the hard disk, the data is available for report presentation from the data-base or via a spreadsheet, which allows users to configure reports themselves providing flexible manipulation and presentation of the logged data. When used for process and sequence control, real time colour mimics can be added for easier operator comprehension of the plant processes under control.

Prices start at £18K for a minimum control system which is related directly to the plant interface requirement.

*For further information contact Drew Scientific at 12 Barley Mow Passage, Chiswick, London W4 4PH. Tel: 01 995 9382*

